# The Extent of Pulmonary Vein Electrical Connections Predicts the Success of Stand‐Alone Pulmonary Vein Isolation in Persistent Atrial Fibrillation

**DOI:** 10.1111/jce.16622

**Published:** 2025-03-10

**Authors:** Matteo Marchetti, Christelle Haddad, Adrian Luca, Mathieu Le Bloa, Cheryl Teres, Giulia Domenichini, Alessandra Pia Porretta, Claudia Herrera, Etienne Pruvot, Patrizio Pascale

**Affiliations:** ^1^ Arrhythmia Unit, Cardiovascular Department, Centre Hospitalier Universitaire Vaudois and University of Lausanne Lausanne Switzerland; ^2^ Arrhythmia Unit, Louis Pradel Cardiovascular Hospital, Hospices Civils de Lyon Lyon France; ^3^ Arrhythmia Unit, Hôpital Bichat Claude‐Bernard, Assistance Publique Hôpitaux de Paris Paris France

**Keywords:** catheter ablation, persistent atrial fibrillation, pulmonary vein activity, pulmonary vein connection, pulmonary vein isolation

## Abstract

**Background:**

Identification of persistent AF (PsAF) patients who may be cured with stand‐alone pulmonary vein isolation (PVI) would allow more efficient ablation strategies. Intuitively, the benefit of PVI is expected to be lower when PVs are poorly connected, but this assumption has never been tested.

**Objective:**

Evaluate whether the circumferential extent of PV connections assessed pre‐ablation predicts the success rate of PVI.

**Methods:**

Consecutive patients with PsAF undergoing first ablation with stand‐alone PVI were included. The extent of PV electrical connection was assessed using a circular mapping catheter and graded as limited, moderate, or extensive, according to tertiles of the mean numbers of bipoles recording PV potentials along the circumference of each vein.

**Results:**

One hundred and forty‐eight patients were included (age 64 ± 9.7 years, PsAF duration 7.3 ± 7 months). After a mean follow‐up of 38.6 ± 7.8 months, freedom from arrhythmia recurrence after last PVI was significantly lower in patients with limited (38.6%), compared to moderate, and extensive PV connections (69.7% and 69%, respectively; *p* = 0.002). While redo PVI procedures did not significantly affect final ablation outcomes in patients with limited PV connections, a significant incremental arrhythmia‐free survival gain was observed with increasing extent of PV connections (*p* < 0.01).

**Conclusions:**

The circumferential extent of PV connections is correlated to the success rate of PVI in patients with PsAF and may be a marker of the contribution of PVs to AF. The finding of limited PV connections may be used to select patients who may benefit from first‐line adjunctive ablation.

## Graphical abstract

The extent of pulmonary vein electrical connection predicts the success of pulmonary vein isolation in patients with persistent atrial fibrillation.

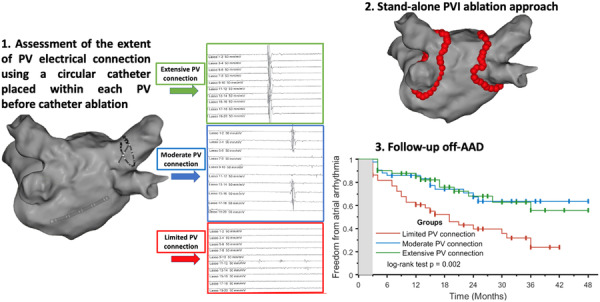

**Abbreviations**
AAD: antiarrhythmic drug
CTI: cavo‐tricuspid isthmus
EGM: electrogram
FU: follow‐up
IQR: interquartile range
LA: left atrium
PsAF: persistent AF
PV: pulmonary vein (LSPV; left superior PV. LIPV; left inferior PV. RSPV; right superior PV and RIPV; right inferior PV)
PVI: pulmonary vein isolation
RFCA: radiofrequency catheter ablation

## 1 Introduction

Pulmonary vein isolation (PVI) is the current cornerstone radiofrequency catheter ablation (RFCA) strategy for AF, since pulmonary veins (PVs) play a major role in the initiation and maintenance of AF [1]. However, while PVI is particularly effective among patients with *paroxysmal* AF, it carries lower success rates in *persistent* AF (PsAF) [2]. Recurrence rates of nearly 50% have been reported in meta‐analyses of stand‐alone PVI, notwithstanding the inclusion of a low proportion of patients with structural heart disease and long‐standing PsAF [3, 4, 5]. In these patients, the mechanism of AF is likely independent of the PVs, and additional ablation strategies targeting the atrial substrate or extra‐PV triggers are likely required. Considering resource utilization and the potential risks of more extensive ablation strategies such as the creation of areas of iatrogenic arrhythmogenesis [6, 7], discriminating these patients from those who may be cured by stand‐alone PVI might allow a more efficient patient‐tailored ablation strategy and help predict outcome.

The observation of overall poorly connected PVs during pre‐ablation mapping is intuitively regarded as a marker of poor response to PVI alone. However, this common conception has, to our knowledge, never been formally evaluated. We, therefore, aimed to study whether the extent of PV connections assessed during routine mapping with a circular catheter before ablation may provide a clue to the contribution of the PVs to the mechanism of PsAF, and thereby predict the success rate of a stand‐alone PVI ablation strategy.

## 2 Methods

### 2.1 Study Population

Consecutive patients with symptomatic PsAF who underwent first‐time RFCA were considered for inclusion. Patients with uninterrupted spontaneous longest AF episode lasting at least 1 month and who underwent a complete mapping of all PVs with a circular catheter before RFCA were included. To evaluate the contribution of the PVs to the mechanism of AF, only patients in whom the ablation strategy consisted of a stand‐alone PVI approach, without adjunctive substrate‐based or non‐PV trigger ablation, were included.

Patients with the following criteria were excluded: (i) any previous RFCA procedure except for cavo‐tricuspid isthmus (CTI) ablation, (ii) history of a non‐CTI‐dependent atrial flutter, (iii) follow‐up (FU) duration shorter than 6 months following a postprocedural blanking period of 3 months (i.e., minimal total FU of 9 months following index PVI).

Our study was approved by an institutional ethical review board (Cantonal Ethics Committee Vaud, CER‐VD) and all patients provided written informed consent.

### 2.2 Electrophysiological Study and Ablation Procedure for PsAF

All patients were on oral anticoagulation for ≥ 1 month before the procedure. Antiarrhythmic drugs (AAD) were continued. Preprocedural transesophageal echocardiography was performed to exclude atrial thrombus. Surface electrocardiograms and bipolar intracardiac electrograms (EGMs) were monitored continuously and stored on a computer‐based digital amplifier/recorder system (Axiom Sensis XP, Siemens, Berlin, Germany). Bipolar intracardiac EGMs were recorded at a sampling rate of 1 or 2 kHz (band‐pass filtered 30–300 Hz, 50 Hz notch filtered). The procedure was carried out under conscious sedation or general anesthesia using the CARTO‐3 electroanatomical mapping system (Biosense Webster, Diamond Bar, California). A 22‐pole variable circular Lasso catheter (Lasso 2515 NAV eco Variable Catheter, Biosense Webster, Diamond Bar, CA) with closely spaced electrodes (2‐6‐2 mm) was used to map the PVs and left atrium (LA). Irrigated tip ablation catheters with contact force‐sensing technology (Thermocool SmartTouch and SmartTouch SF, Biosense Webster, Diamond Bar, California) were used for ablation. The circular mapping catheter was placed sequentially within each PV before ablation to record PV potentials and assess the extent of PV electrical connection. Wide antral circumferential PVI was performed with point‐by‐point applications in temperature‐limited power control mode guided by automatic ablation annotation, minimum ablation index values, local EGMs, and impedance changes. Isolation of the PV antrum was considered complete when all PV potentials within each antrum were abolished as recorded by the circular mapping catheter. Patients remaining in AF at the end of the procedure were electrically cardioverted back to sinus rhythm (SR) to confirm the antral entrance block at the PVs. The waiting period after PVI was left at the discretion of the operators. As per our institutional protocol, a PVI‐only ablation strategy was adopted for all patients with PsAF.

### 2.3 Follow‐up After PVI

AADs were discontinued within 3 months after the first PVI in the absence of a continued indication. After the procedure, patients were evaluated at 3, 6, 9, 12 months with ambulatory 24–48 h monitoring either at our center or by their referring cardiologist. In case of freedom of atrial arrhythmia recurrence 12 months after first PVI, patients were seen by their referring cardiologist every 6 to 12 months.

In the event of arrhythmia recurrence beyond the initial 3‐month blanking period, patients were offered a repeat procedure. When PV reconnections were identified, the same PVI‐only strategy was used for the repeat procedure. In the case of additional ablation strategies (except for CTI ablation), censoring of the subsequent FU ensued.

### 2.4 Clinical Outcome

Success after first PVI was defined as maintenance of SR (absence of any sustained atrial arrhythmia) during FU after AAD cessation (off‐AAD), with a postprocedural blanking period of 3 months. Success after the last procedure was defined as maintenance of SR following last ablation with or without AAD with a post‐procedural blanking period of 3 months.

### 2.5 Assessment of the Extent of PV Electrical Connection

The assessment of the extent of PV connections was based on the analysis of the circular mapping catheter EGMs once positioned near the ostium of the PVs, but distally enough to record clearly discernible PV potentials. The extent of the circumferential PV electrical connection was graded by the operator during the procedure, ensuring blinding to the subsequent ablation outcome. The extent was graded in tenths of PV circumference for each vein, based on the number of bipoles recording distinct PV potentials. Based on the tertiles of the sum of all patients' mean grading of PV connections, the extent of PV connections was qualitatively categorized as limited, moderate, and extensive.

### 2.6 Statistics

Continuous variables are presented as mean ± standard deviation (SD) or median and interquartile range (IQR). Kolmogorov‐Smirnov test was performed to assess variable distributions. Continuous variables displaying nonparametric distribution were compared with the Kruskal–Wallis ANOVA test. Categorical variables are summarized as frequencies and percentages and were compared with Fischer's exact test. The AF‐free survival curves for patient subgroups depending on the extent of PV electrical connection were estimated by the Kaplan–Meier method. Differences between curves were tested with the log‐rank test. Survival curve pairs were compared using the area between their survivals computed on a 6‐month sliding window. All tests were two‐tailed and statistical significance was considered for *p* < 0.05. Statistical analysis was performed using Graphpad Prism (Graphpad Software, Boston, MA, USA) and Matlab (Mathworks Inc., Natick, MA, USA).

## 3 Results

### 3.1 Study Population

Among 170 consecutive patients who underwent a first PVI for PsAF lasting > 1 month, 148 met the inclusion criteria and were studied. Thirteen patients were excluded because of incomplete circumferential mapping of all PVs, one because of a history of a non‐CTI‐dependent atrial flutter and eight because of adjunctive ablation lesion sets beyond PVI. In five of these eight cases, complementary ablation after PVI was dictated by the identification of an extra‐PV trigger during the procedure. Therefore, in only three cases (2%), additional lesion sets were performed because the electrophysiologists in charge felt that PVI alone would probably not be sufficient. Mean age was 64 ± 9.7 years and 73% were male. Median CHA_2_DS_2_‐Vasc score was 2 (IQR: 1.25–3) and the mean duration of the longest continuous AF episode was 7.3 ± 7 months. At index PVI, about half of the patients were in AF at the beginning of the procedure (51%) and/or on amiodarone (47%). Baseline clinical characteristics of the study population are presented in Table 1.

**Table 1:** Clinical characteristics.

	Overall (*n* = 148)	Extent of PV connections			*p*‐value
		Limited (*n* = 50)	Moderate (*n* = 49)	Extensive (*n* = 49)	
*Demographics*					
Age, mean (±SD), years	64 (10)	66 (10)	64 (10)	62 (9)	0.09
Gender M/F, *n* (%)	108 (73)/40 (27)	29 (58)/21 (42)	37 (76)/12 (24)	42 (86)/7 (14)	0.01
Hypertension, *n* (%)	105 (71)	37 (74)	34 (69)	34 (69)	0.84
Body mass index, median (IQR), kg/m^2^	28 (25–33)	28 (24–31)	29 (26–33)	30 (26–33)	0.16
Sleep apnea syndrome, *n* (%)	55 (37)	15 (30)	18 (37)	22 (45)	0.30
History of smoking, *n* (%)	37 (25)	12 (24)	11 (22)	14 (29)	0.76
Diabetes, *n* (%)	10 (7)	3 (6)	2 (4)	5 (10)	0.46
Number of AAD used, median (IQR)	2 (1–2)	2 (2–2)	2 (1–2)	2 (1–2)	0.53
Amiodarone, *n* (%)	69 (47)	27 (54)	18 (37)	24 (49)	0.46
*AF history*					
History of AF, mean (±SD), years	3.7 (3.9)	3.8 (4.3)	3.8 (3.7)	3.5 (3.7)	0.91
Uninterrupted AF duration, mean (±SD), months	7.3 (7)	8.3 (8.2)	6.9 (5,9)	6.6 (6.7)	0.35
*Structural heart disease*					
Tachycardiomyopathy, *n* (%)	62 (42)	22 (44)	19 (39)	21 (43)	0.85
Valvular disease, *n* (%)	31 (21)	14 (28)	11 (22)	6 (12)	0.14
Coronary artery disease, *n* (%)	16 (11)	7 (14)	6 (12)	3 (6)	0.41
*Stroke risk factors*					
CHA_2_DS_2_‐VASc score, median (IQR)	2 (1.25–3)	3 (2–3)	2 (1–3)	2 (1–3)	0.07
*Cardiac parameters*					
LVEF, median (IQR), %	60 (53–65)	60 (53–66)	60 (54–63)	58 (52–63)	0.53
LA volume index, median (IQR), mL/m^2^	39 (34–47)	41 (34–50)	38 (33–47)	39 (30–45)	0.48
LA diameter, median (IQR), mm	47 (41–52)	47 (40–53)	45 (42–50)	46 (41–52)	0.67

Abbreviations: AAD, antiarrhythmic drugs; AF, atrial fibrillation; IQR, interquartile range; LA, left atrium; LVEF, left ventricular ejection fraction.

### 3.2 Characterization of the Extent of PV Connections

**Figure 1:**
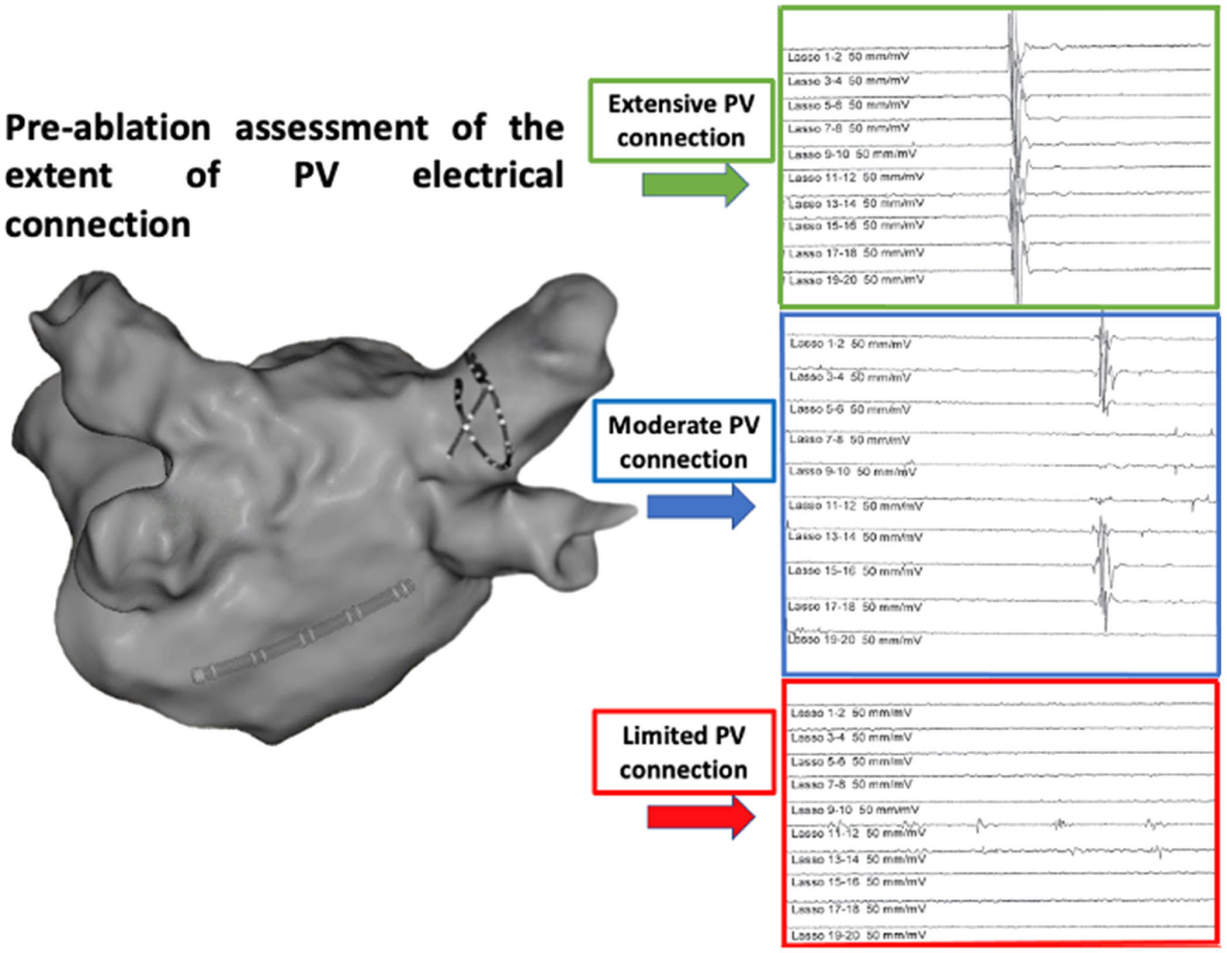
Grading of the circumferential extent of PV connections. Tracings showing examples of extensive (green, 100%), moderate (blue, 60%) and limited (red, 10%) PV connections.

Overall, the mean degree of PV connection was 61 ± 27% (LSPV 63 ± 28%, LIPV 63 ± 27%, RSPV 53 ± 27%, RIPV 64 ± 26%). Based on tertiles of the mean percentage of PV circumference displaying PV potentials, the extent of PV connections was qualitatively characterized as limited (*n* = 50), moderate (*n* = 49), and extensive (*n* = 49), based on a mean percentage of the PV circumference displaying PV potentials of ≤ 55%, > 55% to 70% and > 70%, respectively. Illustrative examples of the PV connections extent grading are shown in Figure 1. Except for gender, no significant differences were observed in baseline characteristics among patients with limited, moderate, or extensive PV connections (Table 1). A significantly lower proportion of women was observed in patients with extensive PV connections compared to patients with limited PV connections (14% vs*.* 42%, respectively; *p* = 0.01). Overall, the median extent of PV connections was significantly lower among women than men (51% vs. 65%, *p* = 0.007).

### 3.3 Clinical Outcome After PVI and Extent of PV Connections

**Figure 2:**
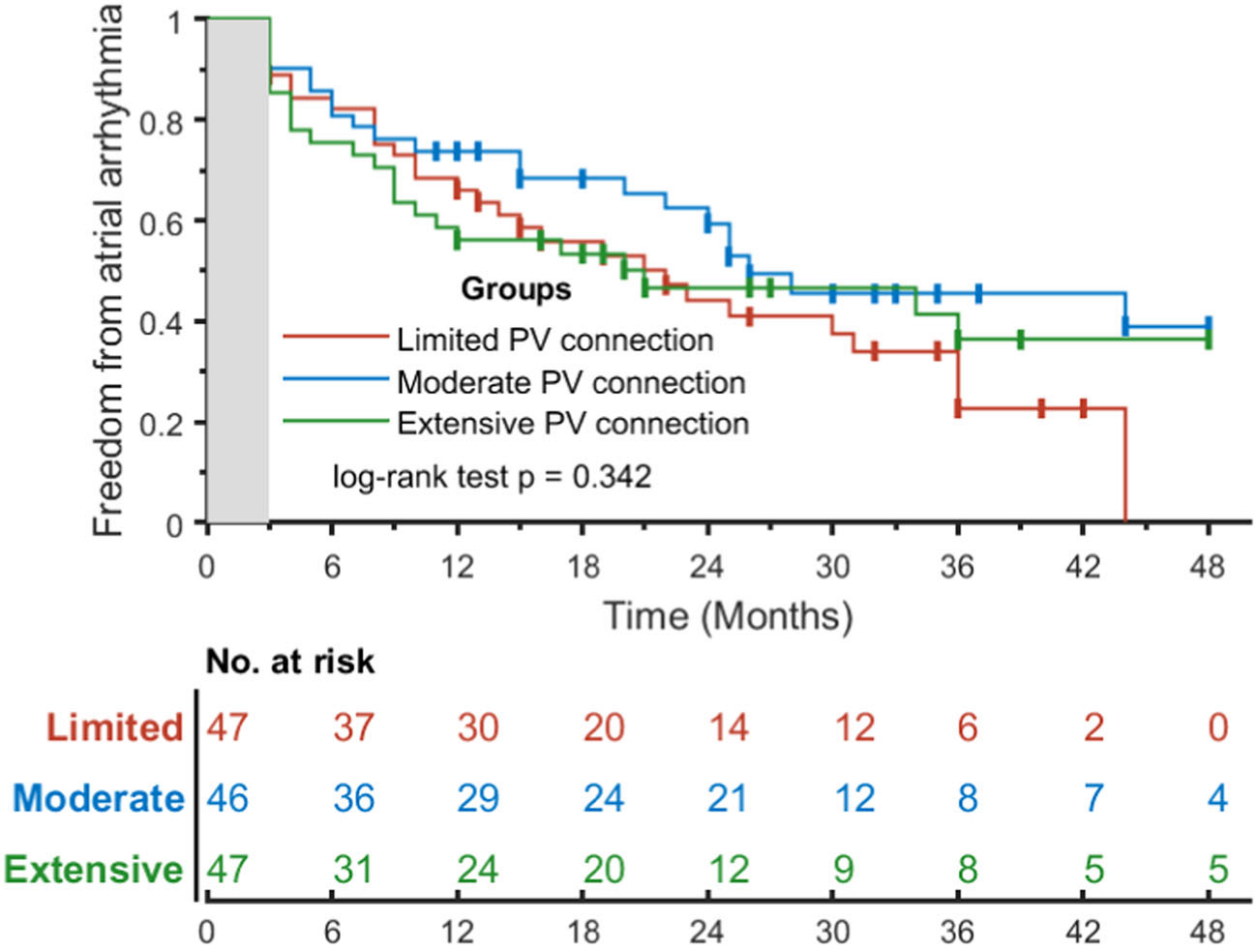
Kaplan–Meier arrhythmia‐free survival curves after first PVI‐only procedure off‐AAD. Patients are stratified according to the extent of PV connections. PVI, pulmonary vein isolation; AAD, antiarrhythmic drug.

Two patients were lost to FU after the initial procedure. After a mean total FU of 38.6 ± 7.8 months, freedom from arrhythmia recurrence after a single ablation procedure off‐AAD was achieved in 54 patients (42%). After first PVI, freedom from arrhythmia recurrence off‐AAD in patients with limited, moderate, or extensive PV connections was achieved in 35.6% (*n* = 16), 50% (*n* = 21) and 41.5% (*n* = 17) of patients, respectively (*p*‐value 0.342) (Figure 2). Among patients who presented with recurrent arrhythmia (*n* = 86), 51 were in PsAF (59%), 28 were in paroxysmal AF (33%), and 7 (8%) in atrial tachycardia. Among patients with extensive PV connections, paroxysmal forms of AF were the most frequently observed recurrent arrhythmia, whereas a minority of patients with limited extent of PV connections presented a recurrence in paroxysmal AF (55% vs*.* 19%; *p* = 0.005).

A repeat ablation procedure was performed in 78 out of 86 patients with arrhythmia recurrence after the index PVI. Durable PVI was observed in 17 patients (22%). Six patients underwent > 2 procedures. After a mean of 1.4 ± 0.6 procedures and a mean follow‐up of 25.2 ± 7.8 months from the last procedure, the overall success rate was 72.9%, including 26.7% on AADs (*n* = 28).

**Figure 3:**
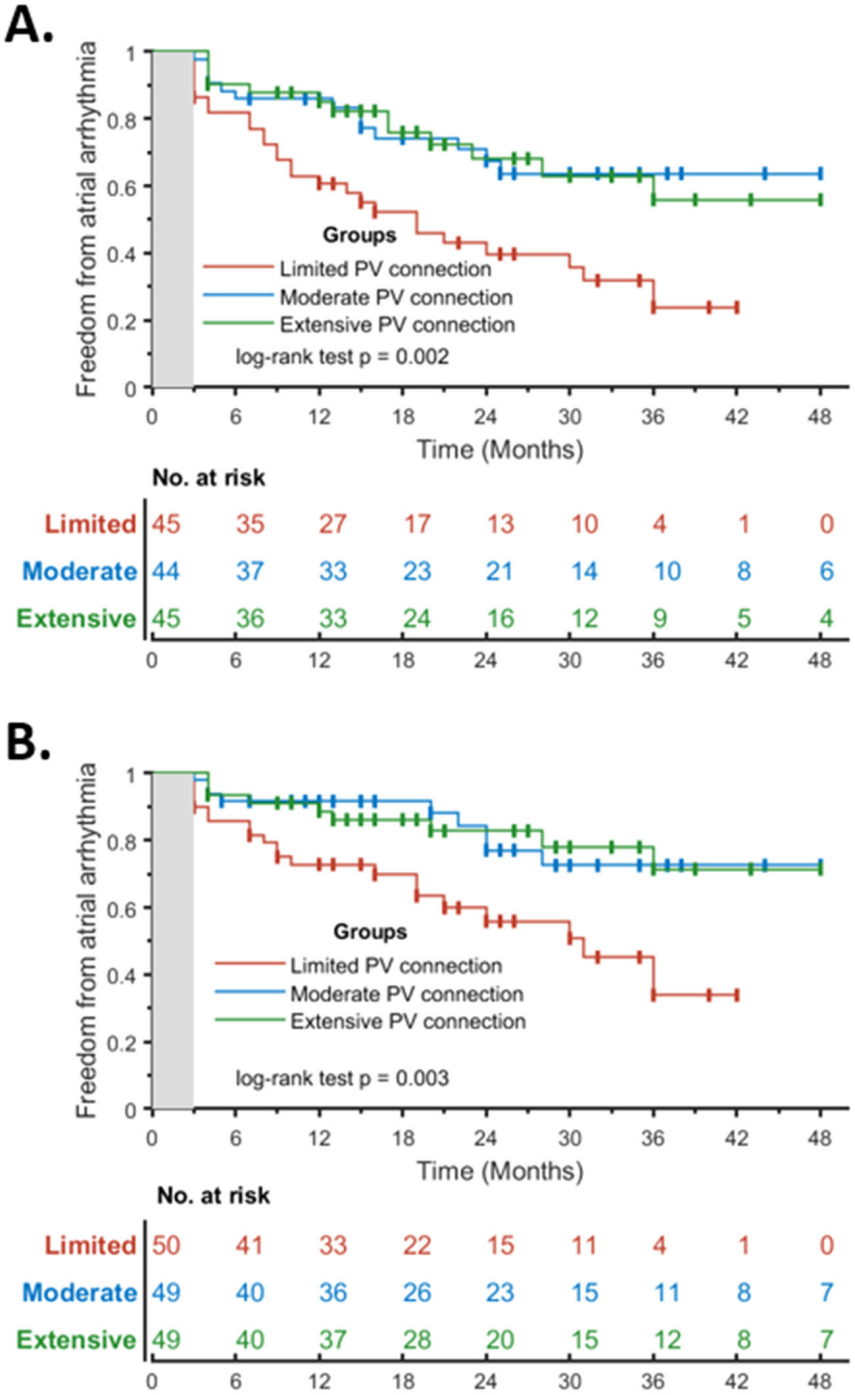
Kaplan–Meier arrhythmia‐free survival curves after last PVI‐only procedure for the population off‐AAD (A), and the entire population (B). Patients are stratified according to the extent of PV connections. PVI, pulmonary vein isolation; AAD, antiarrhythmic drug.

After the last procedure, freedom from arrhythmia recurrence on or off‐AAD at the end of FU in patients with limited, moderate, or extensive PV connections was achieved in 57.1% (*n* = 28), 81.3% (*n* = 39) and 80.9% (*n* = 38) of patients (*p*‐value 0.003). Similarly, the off‐AAD success rate was 38.6% (*n* = 17) in patients with limited extent of PV connections, compared to 69.7% (*n* = 30) and 69% (*n* = 29) in patients with moderate or extensive PV connections, respectively (*p*‐value of 0.002). Figure 3A and B display Kaplan–Meier arrhythmia‐free survival curves after the last procedure stratified according to the extent of PV connections.

Ten patients presented at least one spontaneously isolated PV during initial mapping (7%). A trend towards a lower success rate of PVI after the last procedure off‐AAD was found in these patients compared to those without any spontaneously isolated PV (30% vs*.* 61.3%, respectively; *p*‐value = 0.061).

### 3.4 Incremental Value of Redo PVI According to the Extent of PV Connections

**Figure 4:**
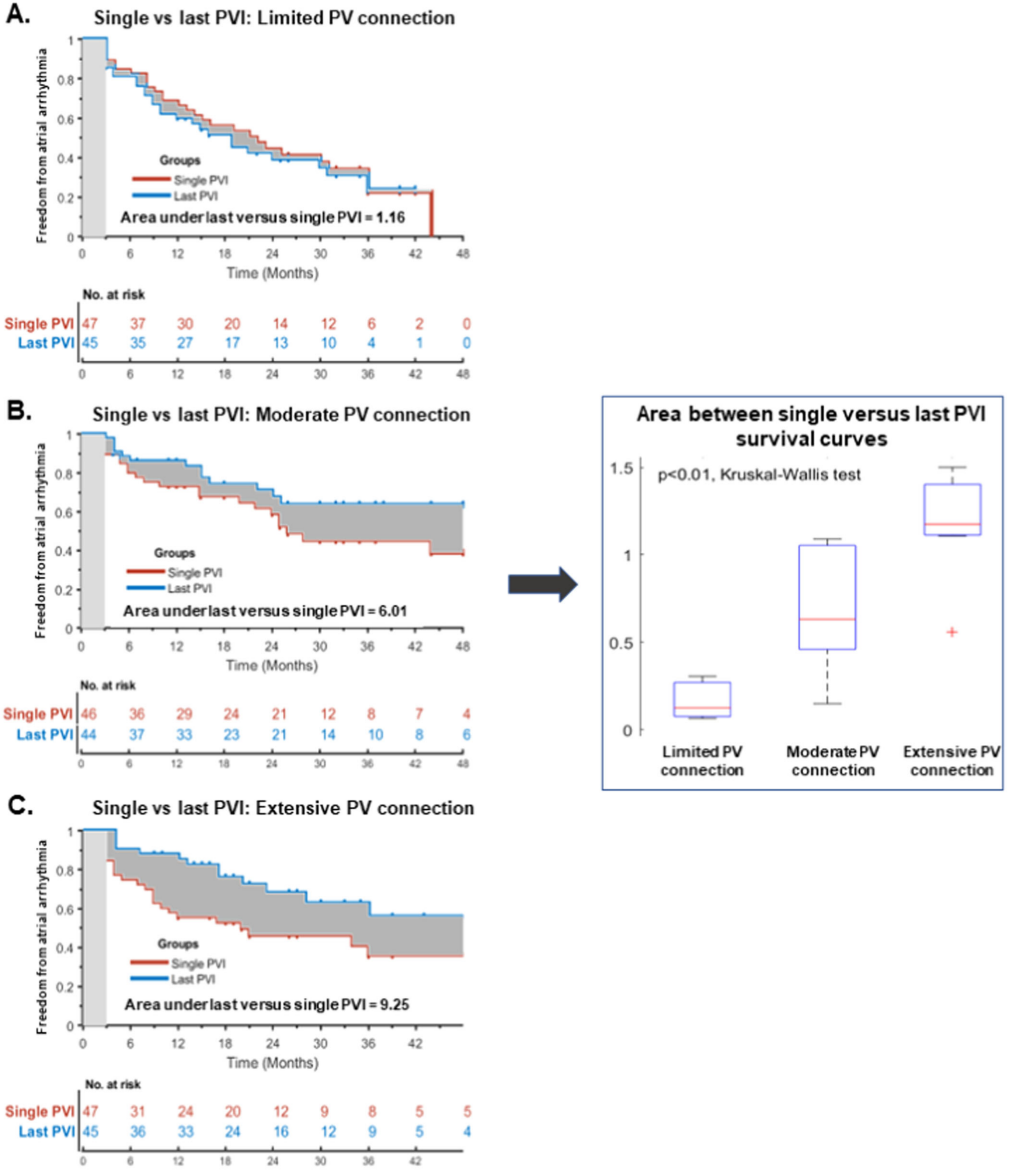
Left, Kaplan–Meier curves showing the arrhythmia‐free survival gain between single versus last PVI‐only procedures in patients with limited (A), moderate (B), and extensive (C) PV connections. Right, Boxplot of the area under *last* vs. *single* PVI survival curves showing an incremental arrhythmia‐free survival gain with increasing extent of PV connections. PVI indicates pulmonary vein isolation.

The analysis of the arrhythmia‐free survival gain obtained comparing single versus last PVI procedures, demonstrated that redo PVI did not significantly affect final ablation outcomes in patients with limited PV connections. On the other hand, redo PVI provided an incremental arrhythmia‐free survival gain in patients with increasing extent of PV connections, as illustrated in Figure 4. Accordingly, the area under *last* vs. *single* PVI survival curves increased from 1.16, to 6.01 and 9.25, for patients with limited, moderate, and extensive PV connections, respectively (*p* < 0.01). In patients with extensive PV connections, the absolute arrhythmia‐recurrence risk reduction after last PVI compared to single PVI was 27.6% (95% CI: 7.0%–48.1%) compared to 3.1% (95% CI: −17.0%–23.1%) in patients with limited PV connections.

## 4 Discussion

### 4.1 Main Findings

The present study evaluated whether in PsAF the extent of PV electrical connection can provide clues to their contribution to the mechanism of AF. The major finding is the correlation between the circumferential extent of PV connections and the success rate of a stand‐alone PVI ablation strategy in PsAF. Supporting this observation, we also found that among patients with arrhythmia recurrence: (i) redo PVI procedures did not significantly affect final ablation outcomes in patients with limited PV connections, while redo PVI provided an incremental arrhythmia‐free survival gain with increasing extent of PV connections and, (ii) patients with extensive connections most often recurred in paroxysmal AF while patients with limited connections recurred in PsAF.

### 4.2 Extent of PV Connections as a Marker of the Role of PV Implication in the Mechanism of PsAF

The underlying mechanism of PV arrhythmogenesis in PsAF remains unclear. Previous studies provided evidence that the sleeves of LA myocardium extending onto the proximal portion of PVs may be either the site of origin of triggered activity or serve as a substrate for micro reentry [8, 9, 10, 11, 12, 13, 14]. Both mechanisms may as well be operative, such as the extrasystole that initiates reentry in AF, which may result from triggered firings. Surprisingly, despite the importance of PV sleeves in the pathogenesis of AF and as a therapeutic target for ablation, their variability in extension and electrophysiologic properties has been poorly studied, especially in PsAF patients. Histological studies have shown that PV sleeves are highly variable, with complex muscle bundle orientation and interspersed fibrous tissue. These venoatrial junction variations may either result in continuous circumferential, or in broad vs. discrete discontinuous LA–PV electrical connection [15, 16, 17].

The hypothesis tested in the present study is based on the simple assumption that the lower the extent of PV electrical connection, the more limited the substrate for reentry (or triggered activity) and, since the bulk of benefit is obtained from isolating PVs, the lesser the expected benefit of a stand‐alone PVI procedure in patients with PsAF. Even though many operators share this view, to the best of our knowledge, no study has formally evaluated this hypothesis in PsAF patients so far. Our study fills this gap by providing strong evidence that the contribution of PVs to the mechanism of PsAF is related to the extent of their connections to the LA. Tertiles characterizing the extent of PV electrical connection were correlated to the success rate of a stand‐alone PVI in PsAF patients. The extent of electrically active PV sleeves was graded using a standard circular mapping catheter as it would be performed visually in real‐time routine clinical practice. Differences in outcome with stand‐alone PVI were mainly observed between patients with *limited* PV connections compared to patients with moderate and extensive PV connections, who both shared similar arrhythmia‐free survival. Accordingly, an average connection among all PVs covering about half or less of the PV circumference identifies a subgroup of patients with poor response to a stand‐alone PVI ablation strategy.

The causal relationship between the extent of PV connections and the role of PVs in the pathogenesis of AF is further supported by two concordant observations. First, in patients with limited PV connections, the ablation outcome was not significantly affected by redo PVI procedures as opposed to other patients in whom, after redo PVI, a proportional increase in the arrhythmia‐free survival was observed with increasing extent of PV connections. Second, among patients with arrhythmia recurrence after a single procedure, paroxysmal rather than persistent AF was observed in most patients with extensive PV connections. On the other hand, recurrences as persistent AF were observed in most patients with limited PV connections. These modes of recurrence tend to suggest a trigger‐based mechanism of AF in the former, and a substrate‐based mechanism for the latter [18].

### 4.3 Relation Between the Extent of PV Connections and the Mechanism of AF

The fact that the PV firing that triggers paroxysmal AF episodes may be related to the extent of PV connections has been previously evaluated by Nakagawa et al. [19]. The authors measured each LA–PV connection in tenths of PV circumference based on the number of continuous bipolar lasso electrode sites required for ablation. They demonstrated that the occurrence of isoproterenol‐induced PV firing was related to the extent of LA–PV connections. PV firing was most common in PVs with “circumferential” connections (i.e., along 90%–100% of the PV circumference), and was also more frequent in PVs with “broad” connections compared to PVs with “discrete” connections (i.e., along 40%–80% and 10%–30% of the PV circumference, respectively). Whatever the mechanism of PV firing (microreentry or triggered activity), these findings tend to confirm that the substrate for PV arrhythmogenesis is related to the extent of PV connections. Another study by Babak et al. [20] demonstrated that in patients with PsAF, the presence of local PV capture after cryoballoon ablation identified a subgroup of patients with favorable clinical outcomes following PVI alone (performed during index and any required repeat procedure). Since local capture within the PVs likely reflects greater PV sleeve mass, these findings further support the hypothesis linking the extent of PV connections to the response to PVI in PsAF.

### 4.4 Gender Difference in the Extent of PV Electrical Connection?

Somewhat unexpectedly, a significantly lower extent of PV connections was observed among women compared with men. This observation is, however, in line with a previous report by Ellis et al. who sought to define the association between sex and other clinical characteristics on PV sleeve length assessed using electroanatomic voltage mapping [21]. They observed that among 63 patients with AF and after adjustment for covariates, male sex was associated with a 6.6 mm increase in sleeve length (*p* < 0.001). Gender differences in the contribution of PVs in the pathogenesis of AF is further supported by the fact that women have more arrhythmia recurrences after PVI [22, 23, 24], and have more LA fibrosis [25]. Furthermore, they tend to have fewer PV reconnections at redo ablation [26], and more non‐PV triggers with less arrhythmogenic PVs [26, 27, 28, 29] than men. Based on this body of evidence, the gender difference in the extent of PV connections observed in our study may therefore reflect the fact that, in women, the mechanism of PsAF is more often dependent on adverse remodeling and/or extra PV triggers.

### 4.5 Study Limitations

The main limitation of the study is related to the assessment of the extent of electrical connection between PVs and the LA which may be dependent on the position depth of the circular catheter within the vein, and the potential overlap of bipoles in smaller diameter veins. Nevertheless, the systematic use of a 3‐D mapping system allowed to minimize this source of error. Moreover, the selected methodology implied a subjective bias in the assessment that was minimized by the fact that different operators collected the data. This methodology was selected considering that the study aim was to provide a simple qualitative method that reproduces the operator assessment *during* the procedure and may be easily implemented in routine clinical practice to predict the outcome of PVI only. This was based on the fact that the decision to perform a PVI plus strategy must be taken at that moment. Moreover, it offers the advantage of integrating the observations made during a procedure that may influence the operator assessment, such as the assessment of the far‐field signals recorded in the vein, particularly in AF. As some authors [30, 31] show, high‐resolution/density mapping may allow a more precise characterization of the extent and patterns of PV–LA connections. Further studies correlating such ultrahigh‐resolution preoperative mapping of PVs with the success rate of a PVI‐only approach in PsAF patients are therefore needed. Finally, excluding patients who underwent ablation beyond PVI at first intervention could have introduced a possible selection bias. However, this represented less than 5% of the cases, and for most of them, the additional ablation was indeed motivated by identifying an extra‐PV trigger.

### 4.6 Clinical Implications

Our study provides a readily applicable method to discriminate in routine clinical practice which patients with PsAF may be cured by a PVI‐only approach, from those who may need additional ablation approaches considering very limited success rates otherwise. This would allow for a more efficient patient‐tailored ablation strategy, considering that the indiscriminate use of adjunctive ablation strategies failed to prove beneficial consistently, bears additional risks and is less efficient in resource utilization. Based on our results, identifying poorly connected PVs during index PsAF ablation should prompt the consideration of additional ablation strategies beyond PVI. Additional lesion sets, such as posterior wall isolation or linear ablation, with or without Marshall vein ethanol ablation, would likely best be applied selectively in PVI non‐responders. These patients may be identified based on an average connection among all PVs covering half or less than half of the PV circumference. For the remaining patients, stand‐alone PVI should be favored.

## 5 Conclusions

The success rate of a stand‐alone PVI ablation strategy in PsAF is quantitatively related to the extent of PV electrical connection. This finding may provide the key to develop a patient‐tailored ablation strategy based on the identification of patients that are likely PVI‐non responders and candidate for additional ablation strategies.

## Disclosure

The authors have nothing to report.

## Supporting information

Supporting information.

## Data Availability

The data that support the findings of this study are available from the corresponding author upon reasonable request.
