# Supplementary material for: The Extent of Pulmonary Vein Electrical Connections Predicts the Success of Stand‐Alone Pulmonary Vein Isolation in Persistent Atrial Fibrillation
Source: J Cardiovasc Electrophysiol. 2025 Mar 10;36(5):978–87. doi: 10.1111/jce.16622 (PMC12075904; doi:10.1111/jce.16622)

**Supplemental figure 1.** Illustrative examples of PV connections extent grading: limited PV connection framed in red, moderate PV connection framed in blue and extensive PV connection framed in green.


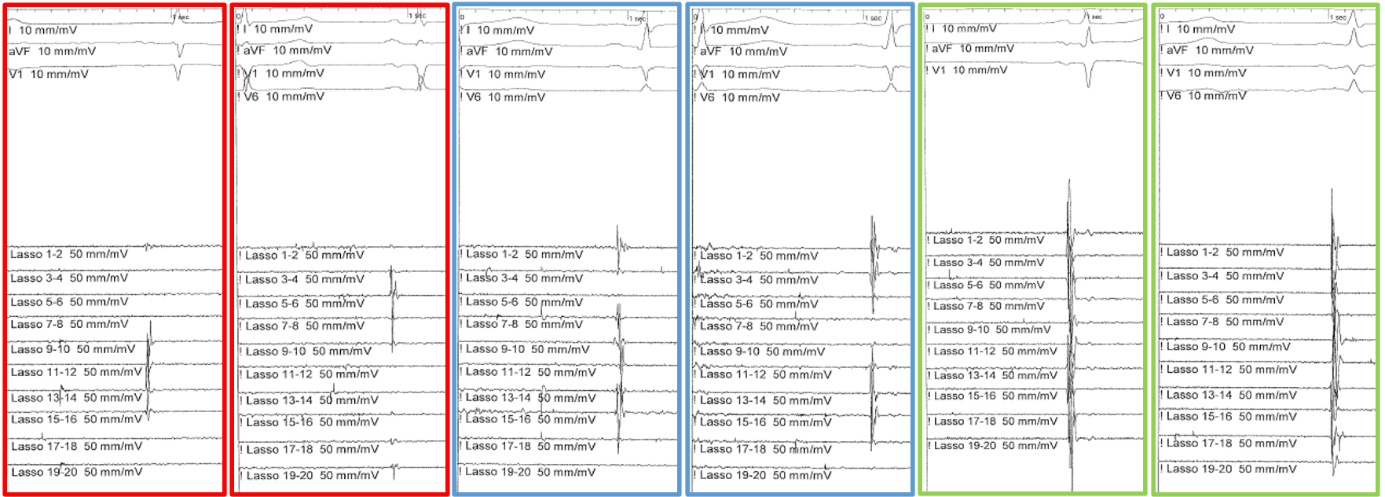

Supplement: Supplementary file 1 — Supporting information. [file JCE-36-978-s001.docx]
